# On the Diagnosis of Hernia

**Published:** 1830-04-01

**Authors:** 


VIII.

ON THE DIAGNOSIS OF HERNIA.

How much has been written, and how much remains for us to write on the subject of hernia, that bug-bear for the young, and too frequently stumbling-block for the old and the experienced. Some good observations on this subject, placed as it is on the confines of medicine and surgery, are contained in the late edition of Dr. Monro's valuable, but too diffuse work. We are not about to enter into the minutiæ or the ordinary points of hernia, God forbid! but we shall glance at one or two interesting portions of the chapter devoted to its consideration. And first of—

INGUINAL HERNIA.

We are all aware that males are peculiarly prone to this, and the other sex to the femoral hernia. The following tables, however, shew on a large and convincing scale the general laws respecting the individuals affected by the disease, the side of the body on which it is most frequent, and its more ordinary complications.

*From July* 6, 1805, *to July* 6, 1811. 1637 *Cases.*

| | | | Males. | Females. |
|---|---|---|---|---|
| 332 double | In both groins, | | 319 | 2 |
| | thighs, | | 0 | 11 |
| 1257 single | Inguinal, | Right side, | 671 | 26 |
| | | Left side, | 354 | 13 |
| | Femoral, | Right side, | 5 | 54 |
| | | Left side | 2 | 37 |
| | In the navel, | | 13 | 76 |
| | Ventral herniæ, | | 3 | 3 |

Several patients having umbilical hernia, had also one or two inguinal or femoral herniæ, one person had a ventral, and two inguinal hernia.*

The following document, extracted from the 25th volume of the Philosophical Magazine, corroborates the former. 3013 patients were examined.

| | | Males. | Females. |
|---|---|---|---|
| 741 Double Ruptures | In both thighs (femoral) | 3 | 44 |
| | In both groins (inguinal) | 609 | 85 |
| 2272 Single Ruptures | In one thigh (femoral) | 57 | 163 |
| | In one groin (inguinal) | 1520 | 399 |
| | In the navel (umbilical) | 36 | 97 |
| | Total, | 2225 | 788-3013 |

Of the single ruptures, more than one-third happened on the left side, and nearly two-thirds on the right side. A very small proportion of triple ruptures, and other extraordinary cases, likewise occurred in the above number; but they were extremely rare, and mostly existed among the female sex.

These statements amply corroborate the general opinions regarding the sex most affected with inguinal or femoral hernia. They likewise prove the much greater prevalence of rupture on the right side than on the left, and the greater disposition in females to suffer from umbilical herniæ. Passing over the common varieties of the disease we stop at—

*Inguinal Herniæ, which in situation resemble Crural Herniæ, owing to Malconformation of the Inguinal Canal.*

The fourth modification of inguinal hernia is very rare, and occasioned by

---

* I am indebted to that distinguished physician Dr. G. GREGORY of London, for this statement."

the malconformation of the inguinal canal, which is owing to the smaller tendinous fibres, which connect the larger columns of the external oblique muscle, being wanting.

In these circumstances the inguinal canal is imperfect; hence the bowels, when protruded, do not follow the course of the inguinal canal, but push immediately downwards and outwards, forming a tumour in the situation of a crural hernia.

This modification of hernia has been described by the late Professor Hamilton of Glasgow, and also by Petit. It remained for the late Mr. Allan Burns of Glasgow to detect the cause of such a deviation, which he has described to me in the following letter, dated March 7, 1806:

The tumour was found in the bend of the left thigh. The inguinal canal was fully as large as it is usually met with in the male, and besides, so very short, that it presented, when fully unfolded, almost the appearance of a mere aperture. The round ligament of the womb was enveloped in a distinct tunica vaginalis, and bearing the same relation to the intestine that the spermatic cord does in the other sex. On the right side, the herniary sac was about two inches in length, and in shape resembled a Florence flask; the bulbous extremity extending from the lower orifice of the canal, was contained in the upper part of the thigh, *lying more in the course of a crural than of an inguinal hernia.*

This deviation from the usual direction of the tumour was produced by a premature separation from each of the external pillars of the inguinal canal. Where the inguinal canal is imperfectly formed, it is generally owing to the incomplete extension of the posterior or internal side of the ring.

Where this happens, the internal orifice of the canal is brought nearer to the pubes than it ought to be, but when the imperfection is produced by a premature separation at the external pillars, then, by dissection, we find the internal orifice in its proper place, but the external outlet is removed from the pubes.

In the first instance, when the herniary tumour protrudes, it lies just over the tubercles of the pubes, and follows the course of the spermatic cord into the scrotum, while in the latter it lies nearer to the spine of the ilium, and is seated just over the crural foramen, and by extension, descends along the thigh, *counterfeiting the appearance of femoral hernia.*

By attention, however, it is readily distinguished from the latter, by being felt lying over the crural arch, and on the outer side of the tubercle of the pubes.

When the bowels follow the course of the inguinal canal, the epigastric artery is situated nearer to the symphysis pubis than the hernia; whereas, when the bowels do not follow such a course, but pass only through the under abdominal aperture, then the epigastric artery is situated nearer to the anterior spinous process of the ilium than the hernia.

The following remarks on the *Affections liable to be confounded with Hernia* are deserving of consideration and attentive perusal.

Swellings in the groin, or in the scrotum, proceeding from various causes, may sometimes bear a strong resemblance to inguinal or scrotal hernia.

1st, Fat in the groin sometimes assumes the shape of an inguinal or crural hernia, and being covered by a thin layer of condensed cellular substance, communicates to the touch nearly the same sensations as an omental hernia. If a patient, with masses of fat so situated, had been seized with colic and vomiting, and had been at the same time much constipated, recourse might have been had to an operation, for pressure does not alter the shape or bulk of such tumours.

The history of the progress of the swelling unfolds the nature of it.

2d, The testis, when arrested in the under abdominal aperture, forms a tumour not unlike to an inguinal hernia, more especially as the testis is not fixed in its place.

A swelling in the groin, originating from such a cause, may be discovered by the want of the testis in the scrotum, and by the very peculiar sensation which pressure on the testis creates.

The case becomes much more puzzling when a turn of the intestine slips down behind the testis, sticking at the groin.

3d, A polypus contained in a cyst growing from the spermatic cord, bears a strong resemblance to an inguinal hernia.

4th, Crural or pudendal hernia has sometimes been mistaken for the inguinal. The diagnosis is founded upon the situation of the neck of the tumour; if the neck be placed above the margin of the crural arch, the tumour is an inguinal hernia, if under it, a crural hernia.

The pudendal hernia, situated in the middle of the labium, may be distinguished from the inguinal hernia by being placed on the inner side of the ramus of the ischium, and may be traced as far as the vagina extends: besides, there is no swelling in the course of the round ligament of the uterus from the groin.

5th, An inguinal hernia bears a considerable resemblance to a hydrocele, especially when the hydrocele extends within the inguinal canal, or when the serous fluid within the hernial sac is in large proportion to the other contents of the tumour, a fluctuation may be perceived in that part of the hernial sac. The fluctuation and pellucidity of the tumour have been supposed to be characteristic of hydrocele; but neither of these symptoms are perceptible in a hydrocele of some duration, from the thickness and tension of the vaginal coat, or from an admixture of coagulated blood with the effused fluid.

The hydrocele of the vaginal coat is an uniform swelling, which begins in the lower part of the scrotum, and gradually extends upwards to the under abdominal aperture: the spermatic cord above the tumour may be perceived of its natural size above, but with difficulty behind the tumour. There is no derangement of the functions of the alimentary canal: and the bulk of tumour, which has an uniform feeling when pressed, does not vary in different positions of the body.

7th, An inguinal hernia may be mistaken for an enlargement of the veins of the spermatic cord.

This kind of tumour, like a hernia, increases in bulk on coughing, becomes much less when the patient lies on his back, and sometimes can be pressed into the abdomen: the swelling begins in the lowest part of the scrotum, and ascends gradually.

The above diseases may be distinguished in the following manner. In both cases, the tumour disappears whilst the patient is in bed. If pressure be made whilst the patient is in that situation, upon a hernia, the tumour does not reappear when the patient gets up; whereas, if the enlarged spermatic veins cause the swelling, in consequence of the pressure impeding the return of the venous blood, and not interrupting the flow of blood by the spermatic arteries, the tumour attains a considerable bulk.

8th, A scrotal hernia which sometimes originates from external violence, may be mistaken for the effusion of blood within the vaginal coat, or within the body of the testis. But in the latter case, the tumour does not become larger upon the patient coughing, is accompanied by sense of weight, and communicates a very peculiar sensation when pressed: the spermatic cord at the under abdominal aperture is not involved in the swelling, and the skin over the tumour has a red colour.

Upon the whole, the inguinal hernia may be distinguished from other swellings at the groin, by attending to the origin and progress of the disease; by observing that the swelling is first perceived at the groin, and that it is observed to pass into the inguinal canal. These marks, combined with the other symptoms already enumerated, characterise the common inguinal hernia.

We are much surprised that encysted hydrocele of the cord has not been enumerated by our author in the foregoing list of diseases likely, or at least liable to be mistaken for inguinal hernia. It is one which has been and frequently is a source of difficulty to surgeons of much experience, and ought certainly to have been mentioned on the present occasion. Dr. Monro might likewise have pointed out the complications of inguinal hernia more at length, we mean its complications with one or more of the foregoing maladies. For instance, we witnessed a case where a small inguinal intestinal hernia was mixed up with varicocele and hernia humoralis. It would be difficult to describe the additional obscurity and complexity thrown on the diagnosis of the case in the first instance by this mixture of diseases. As the *diagnosis* of the complaint is thus a matter of such paramount importance, we pass to that of

### FEMORAL HERNIA.

On account of the very small size and flatness of the tumour in crural hernia, and laxity of the skin and fat, it is often very difficult, by pressure with the fingers, to discover the disease, or to ascertain the nature of the contents of the tumour. Another cause of difficulty is the vicinity of the tumour to the neighbouring lymphatic glands.

"A swelling of the inguinal lymphatic glands bears a very strong resemblance to a crural hernia which contains omentum. Besides, some of the smaller inguinal glands frequently cover the hernial tumour. In both the above cases, the hernial tumour has not its usual characters, elasticity and smoothness of surface.

The hernial tumour commonly appears suddenly after some violent exertion, as after raising a heavy weight, or from a fit of coughing, or vomiting, and increases or diminishes in bulk by pressure, or when the patient goes to bed.

In many cases of crural hernia, the tumour is so small, that an opinion is formed respecting the nature of the disease, rather from the concomitant symptoms than from the appearance of the tumour.

Symptoms of strangulation coming on, remove all doubt respecting the nature of the case.

Notwithstanding these and other marks of distinction, crural hernia has been mistaken for scrofulous enlargement of the inguinal glands, or a venereal bubo, for a hernia."

Our author met with "a puzzling case" at his Dispensary. A girl presented herself with a very painful swelling in the groin which she said had appeared suddenly. As, however, it was not situated exactly over the crural ring, and neither occasioned sickness or costiveness, the nature of the case was determined to be scrofulous. We confess that we see nothing very puzzling in this, unless the diagnosis of ordinary bubo in the groin be considered a matter of some mystery. Certain we are that we never saw a girl so affected who did not *say* that the tumour came suddenly after a blow, or without one. "Getting over a stile" is a constant cause of venereal buboes in the young women who apply at St. George's Hospital. Enlarged glands in the groin have, notwithstanding, been mistaken for hernia by very good surgeons. A laughable exemplification of this blunder occurred, some years ago, in a hospital in this metropolis. The surgeon cut down upon the tumour secundùm artem and with great precision, when all at once an indurated lymphatic gland, released from the pressure of the fascia and its cellular sheath, popped out to the other side of the operation-room, and the hernia was no where to be found. To revert to our author, we are informed that Mr. Else met with a femoral hernia placed behind a swelled suppurating lymphatic gland of the groin. We know ourselves of one or two cases of such complication where the operation was rendered extremely difficult and confused. "The diagnosis becomes still more obscure, if suppuration shall have taken place in the enlarged lymphatic gland, and also where the hernial tumour is composed of several small sacs; or when an empty hernial sac or hydatid covers the more recent hernial tumour, or when the displaced intestine is included in a double sac."

A quantity of fat, or a collection of hydatids on the inner side of the groin, bears some resemblance to a herniary tumour, as the watery fluid within the elastic coats of the hydatid, communicates to the touch nearly the same sensation as a protruded portion of intestine.

In the Museum of the University of Edinburgh, there is a sac, the size of a hen's egg, and containing a quantity of hydatids, which was removed from the upper and outer parts of the thigh.

Lumbar abscess may be very readily mistaken for a crural hernia; as it becomes larger when the person coughs, and also more tense in the erect than in the horizontal posture.

The characteristic symptoms of lumbar abscess are inflammation in the side or loins, followed by symptoms which indicate the formation of purulent matter, viz. pain, increased on motion, with slow and gradual increase of the tumour, the limits of which are not well defined; fluctuation of matter felt, on alternate pressure being made on the loins, or lower part of the abdomen, and upper and inner part of the thigh when the swelling is examined alternately in the erect and horizontal posture.

A crural hernia of a large size, or when reflected upon the crural arch, is often placed obliquely, as such a hernia covers the under abdominal aperture, and its neck being compressed, and somewhat resembling the spermatic cord, it may be mistaken for an inguinal hernia.

A crural hernia is sometimes reflected upwards and outwards, sometimes upwards and inwards.

In order to ascertain the nature of the hernia, it sometimes becomes necessary to press downwards the reflected part of the swelling; and by grasping Poupart's ligament over the crural aperture the herniary tumour may be traced to the crural aperture. But this cannot always be done when the disease has been of some duration; as, during the progress of the disease, the tumour may be retained in its unnatural situation by adhesions.

A circumscribed varix or swelling of that part of the femoral vein which is next to the crural arch expands when the patient coughs, becomes much less in the recumbent, and expands in the erect posture, and hence has been mistaken for a crural hernia, to which it may bear a strong resemblance.

A swelling occasioned by such a cause, may be discovered, by making pressure upon the vein above the crural arch, so as to prevent the return of blood: thus the varix, by distention, is rendered more prominent.

On account of the small size of the crural aperture, and unyielding nature of its parts, considerable pressure is made upon the neck of a crural hernia, which is sometimes succeeded by thickening and induration; and, for a similar reason, the mucous and muscular coats of the displaced intestine have been, in some cases, ulcerated on the side next to the edge of Gimbernat's ligament.

There is also less chance of returning the contents of a crural than of an inguinal hernia, and strangulation more speedily takes place in the former than in the latter; this points out the danger of postponing beyond a few hours the operation after the symptoms of strangulation appear, and when venesection, the warm bath, and a tobacco clyster, have proved of no avail.

The foregoing remarks on inguinal and femoral hernia though containing nothing very novel in detail are yet well worthy the attention even of the experienced portion of our readers. We are convinced that we cannot do a better office to our brethren, than warn them from time to time of the errors to which they are liable in practice, especially in that of surgery. How many a reputation has been injured, nay blasted, by a slip in the diagnosis of hernia!

